# Improved Quantification of MicroPET/CT Imaging Using CT-derived Scaling Factors

**DOI:** 10.21203/rs.3.rs-3612275/v1

**Published:** 2023-11-30

**Authors:** Ayon Nandi, Masayoshi Nakano, James Robert Brašić, Zabecca S. Brinson, Kelly Kitzmiller, Anil Mathur, Mona Mohamed, Joshua Roberts, Dean F Wong, Hiroto Kuwabara

**Affiliations:** Johns Hopkins School of Medicine: The Johns Hopkins University School of Medicine; Janssen Pharmaceutical KK: Janssen Pharma Kabushiki Kaisha; New York University Grossman School of Medicine; The University of Texas Southwestern Medical Center; University of Maryland Medical Center; Johns Hopkins School of Medicine: The Johns Hopkins University School of Medicine; Ain Shams University; Johns Hopkins School of Medicine: The Johns Hopkins University School of Medicine; Washington University in St Louis School of Medicine Mallinckrodt Institute of Radiology; Johns Hopkins School of Medicine: The Johns Hopkins University School of Medicine

## Abstract

**Purpose:**

Combined micro-PET/CT scanners are widely employed to investigate models of brain disorders in rodents using PET-based coregistration. We examined if CT-based coregistration could improve estimates of brain dimensions and consequently estimates of nondisplaceable binding potential (BP_ND_) in rodent PET studies.

**Procedures:**

PET and CT scans were acquired on 5 female and 5 male CD-1 mice with PET and CT scans were acquired on 5 female and 5 male CD-1 mice with 3-[^18^F]fluoro-5-(2-pyridinylethynyl)benzonitrile ([^18^F]FPEB), a radiotracer for the metabotropic glutamate receptor subtype 5 (mGluR5). In the proposed PET/CT (PTCT) approach, the tracer-specific standard volume was dimension-customized to each animal using the scaling factors from CT-to-standard CT coregistration to simplify PET-to-standard PET coregistration (i.e., 3 CT- and 6 PET-derived parameters). For comparison, conventional PET-based coregistration was performed with 9 (PT9) or 12 (PT12) parameters. PET frames were transferred to the standard space by the three approaches (PTCT, PT9, and PT12) to obtain regional time-activity curves (TACs) and BP_ND_ in 14 standard volumes of interest (VOIs). Lastly, CT images of the animals were transferred to the standard space by CT-based parameters from PTCT and with the scaling factors replaced with those from PET-based PT9 to evaluate agreement of the skull to the standard CT.

**Results:**

The PET-based approaches showed various degrees of underestimations of scaling factors in the posterior-anterior-direction compared to PTCT, which resulted in negatively proportional overestimation of radioactivity in the cerebellum (reference region) up to 20%, and proportional, more prominent underestimation of BP_ND_ in target regions down to −50%. The skulls of individual animals agreed with the standard skull for scaling factors from PTCT but not for the scaling factors from PT9, which suggested inaccuracy of the latter.

**Conclusions:**

The results indicated that conventional PET-based coregistration approaches could yield biased estimates of BP_ND_ due to erroneous estimates of brain dimensions when applied to tracers for which the cerebellum serves as reference region. The proposed PTCT provides evidence of a quantitative improvement over PET-based approaches for brain studies using micro-PET/CT scanners.

## Introduction

Molecular imaging via positron emission tomography (PET) [1–7] continues to improve our understanding of the *in vivo* molecular mechanisms of various diseases [3] owing to ever-evolving animal models of disease [7]. This trend highlights the need for robust methodology and validated analysis procedures for accurate quantification of PET outcome variables.

The analytic procedures for molecular imaging are generally established for rodent PET studies focusing on the brain. First, PET images were transferred to a standard space via either 1) PET-based approaches in which individual animals’ PET images were spatially aligned to tracer-specific PET templates[8–12], 2) CT-based approaches in which individual animals’ CT images were spatially aligned to a standard CT[13–15], or 3) MRI-based approaches in which individual animal’s MR images are spatially aligned to a standard MR image[14, 16–21]. Spatial alignment was achieved by rigid body alignment (a.k.a., coregistration) which included 3-linear, 3-rotational, and 3-scaling parameters (9- parameters; PT9[11, 22, 23]) or together with affine transformation (12-parameters; PT12 [8, 24]), or by non-linear warping (a.k.a., spatial normalization [16, 21, 23]). PET frames are transferred to a standard space using respective coregistration parameters. Thereafter, a template of volumes of interest (VOIs) was applied for the generation of time-activity curves (TACs) of regions for the derivation of PET outcome variables such as BP_ND_, and extraction of regional values from functional volumes such as SUV and SUV ratio volumes.

The CT-based approaches could be natural choices for any study done with PET/CT scanners. In fact, CT images [13, 14, 25, 26] that exert steep intensity changes across the skull surrounding the brain are expected to yield accurate estimates of the brain dimensions of individual animals. Nevertheless, the approaches have not been widely employed because the fundamental conditions for the approaches, the maintenance of in-scanner coregistration of PET and CT (i.e., no head motion between the PET and CT data acquisitions) are often violated, and post hoc coregistration could introduce uncertainties to the analyses should the violation occurred. Thus, the first aim of this study was to develop a novel CT-based approach that did not require PET-CT coregistration. The aim was achieved by devising customized tracer-specific PET templates that were adjusted for the dimensions of individual brains using scaling parameters from the CT to standard CT coregistration. The measure removed the scaling factors from the coregistration of individual PET to the tracer-specific PET templates (thus, a 6-parameter fits), in comparison to the 9-parameter fits employed by typical PET-based approaches.

The proposed CT-based approach, denoted as PTCT here due to its hybrid nature, was compared with two published PET-based approaches, PT9 and PT12. Currently, PET-based approaches are predominantly employed [19, 27] over other approaches potentially due to the simplicity (no need for images from other modalities) and the applicability to both PET/CT and PET/MRI scanners. Despite their popularity, the accuracies of PET-based spatial alignment have never been quantitatively established to our knowledge. It is also unclear if dimensions of individual brains could be accurately estimated by PET images which, in comparison to the CT to standard CT coregistration, show tracer-specific radioactivity distribution patterns with various tapering patterns along the brain edges. Although it has been suggested that these accuracies may be equally evaluated using PET/MRI scanners [16, 18, 28–30],no studies specifically evaluated the accuracies of PET-based coregistration against MRI-based coregistration yet. Further, currently PET/CT scanners are often more readily available and less expensive in many research environments [19], thus we chose to focus on a PET/CT based approach here.

Most importantly, it is not known, or has not been examined the degree to which assumingly trivial errors in estimates of brain dimensions could result in noticeable differences in regional BP_ND_ values, the primary PET outcome variable. The primary aims of this study are to understand whether findings of spatial co-registration accuracies and consequential impact on regional BP_ND_ values allow us to conclude that PTCT is a clear improvement over the typical PET-based approaches, PT9 and PT12.

### Procedures

The data used in this paper was derived from previous work by the authors [31–33]. Detailed methods for preparation of animals and imaging procedures are provided in the supplementary information (**Supplemental Materials, Section 1**).

#### Preparation of standard templates, and volumes of interest (VOIs):

**Figure 1:**
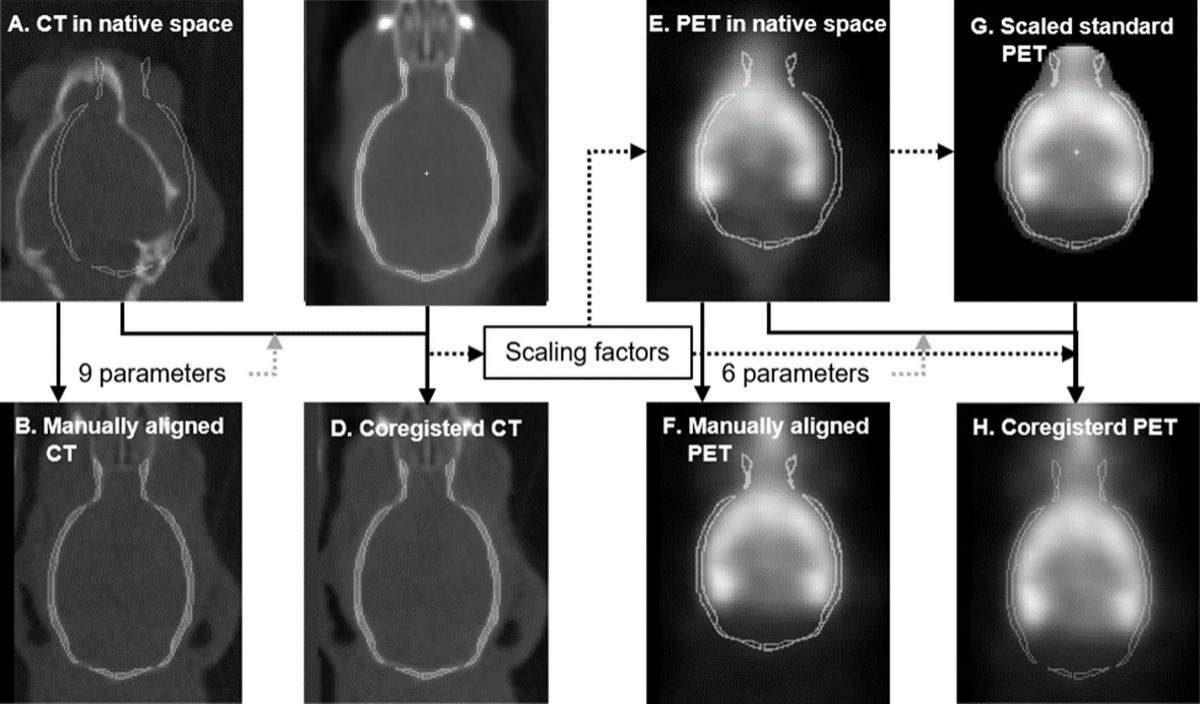
Workflow schemes for coregistration of subject’s CT (**a-d**) and PET (**e-h**) to respective standard volumes. For CT, the 9 parameters from manual registration (B) were refined by SPM12’ coregistration module (**d**). For PET, both standard skull outlines and PET template were rescaled to the dimensions of the subject using the scaling factors from the CT coregistration. The 6 parameters from manual registration (**f**) were refined to transfer PET frames to the standard space (H) incorporating the scaling parameters from the CT coregistration.

The CT and PET templates used for analyses were generated locally by imaging wildtype animals of the same strain (CD-1) mostly commonly used in imaging experiments. The CT template was generated on one animal by co-registering original and left-right flipped volumes. The resulting mid-position CT volume was rotationally adjusted around the left-right such that the cranium and base portions of the skull were in parallel to the dorsal-ventral (DV) axis using the manual registration tool (performed by one of the experienced raters). The PET template volume (a [^18^F]FPEB scan of a wildtype animal) was then aligned using a mid-position PET volume, and rotationally adjusting to fit to the skull outlines of the CT. Individual animals were processed as described above using these initial templates to generate CT and PET volumes in the standard space. The final templates were generated by averaging resulting CT and PET volumes. The MRI template (Fig. 1) was prepared by fitting the mouse MRI atlas developed by the Laboratory of Neuroimaging, University of Southern California (LONI MAP2.0, http://map.loni.usc.edu/) [36, 37] to the skull outlines using the manual registration tool. VOIs for selected brain structures were defined manually on the MRI template using a locally developed VOI definition tool that allowed entering / editing VOIs voxel-by-voxel on three orthogonal views. In a preliminary session, four raters were instructed to refer to the Allen Mouse Brain reference atlas (© 2007 Allen Brain Science Institute, accessed from https://mouse.brain-map.org/static/atlas) [38], while defining VOIs, rotating through the orthogonal views of the template until a consensus was reached (Fig. 1). Left (L) and right (R) hemispheres were defined separately for each of the following regional VOIs: cerebellum (Cb, served as the reference region), cortical mantle (Cx), hippocampus (Hp), hypothalamus (HT), striatum (St), substantia nigra (SN), thalamus (Th), and ventral striatum (Vs).

### Coregistration procedures

**Figure 2:**
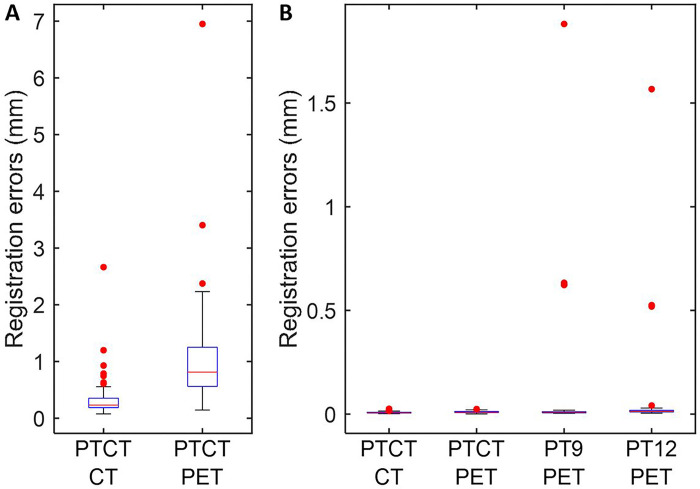
Boxplot of registration errors of initial guesses from individual raters for CT- and PET- portions of PTCT (**a**) and after automated refinements using respective initial guesses (**b**). Note that initial guesses of linear and rotational parameters and scaling parameters from the PET- and CT-portion of PTCT, respectively were used for PT9 and PT12. Red dots indicate statistical outliers (below 2.7 and above 97.3 percentiles).

The proposed PTCT approach consisted of 4 steps including 1) coregistration of CT volumes to the template CT volume via 9-parameter fit (Fig. 1a–d), 2) customization of the PET template volume to the brain dimensions of individual animals using the scaling parameters from Step 1, 3) coregistration of PET volumes to customized standard PET volumes via 6-parameter linear fit (Fig. 1e–h), and 4) transfer of the dynamic PET to the standard space using the 6 parameters from Step 3 and the 3 scaling parameters from Step 1 (Table 1). All coregistration procedures were conducted using the coregistration code of SPM12 (Welcome Department of Imaging Neuroscience, London, UK, www.fil.ion.ucl.ac.uk/spm/).

### Comparison methods

Two published PET-based approaches were employed to compare with PTCT. In the PET-based approaches, the individual PET volumes were directly co-registered to the standard PET, using either 9-parameter (PT9) [11, 22, 23], or 12-parameter affine (PT12) [8, 24] registration.

### Evaluation of the effects of initial visual estimates on spatial registration

The three approaches did not converge on all cases with default initial guesses (0 for linear and rotational parameters, 1 for scaling parameters, and 0 for affine parameters) in a preliminary assessment. To examine dependencies of automated convergence on initial guesses, ten raters manually yielded initial guesses by roughly aligning PET images (for PT9 and PT12) or CT images (for PTCT) to the template skull outlines (Fig. 1d and 1a, respectively) using a locally prepared SPM12-based tool (ten raters completed all CT steps, 8 completed PET). A registration error metric [11, 15, 39, 40] was obtained for each animal as the mean distance from the center points of standard VOIs that were transferred to individual animals’ native PET or CT spaces per raters’ initial guesses (to document inter-rater variations) or their refined estimates to their geographical centers across raters. This metric is analogous to the target registration error metric described in [39, 40]. Automated refinements were considered successful when the registration errors were below one third of the smallest voxel dimensions (i.e., below 0.04 mm for CT and 0.13 mm for PET).

### Quantitative assessment of spatial registration

Observed estimates of parameters were evaluated by differences (PT9 or PT12 less PTCT) for the translational and rotational parameters, and by ratios (PT9 or PT12 over PTCT) for the scaling factors.

#### Quantification of standard uptake values (SUV) and nondisplaceable binding potential (BP _ND_ ):

Time activity curves (TACs) of the regions were obtained by applying the standard VOI set to dynamic PET frames that were spatially transferred to the standard space using observed parameters of PT9, PT12, and PTCT. SUVs of regions were obtained as means of TACs from 30 to 60 min post-injection. Regional values of the non-displaceable binding potential (BP_ND_) were obtained as the target-cerebellum SUV ratios minus one [41].

### Quantitative assessment of scaling parameters

We hypothesized that PET-based coregistration could result in over- or under-estimation of the brain dimensions of individual animals due to errors in scaling parameters. To address this hypothesis, individual animals’ CT images were transferred to the standard space 1) per CT registration parameters from PTCT and 2) using the linear and rotational parameter estimates of CTPT and the scaling factor estimates from PT9. The CT intensity profiles (defined by quantifying CT intensity within a specific region) of individual animals from the two approaches were compared to the standard CT at 5 points where the standard skull was relatively perpendicular to the axes. Note that the anterior end was not included due to the lack of apparent perpendicular points. The approach that showed a better agreement of profiles was considered to yield more accurate scaling parameters. Separately, mean CT intensities in the skull VOI (Fig. 1a–d) were compared between the two approaches. The approach that showed the higher mean intensities was considered to have better alignment of the skull across animals within the skull VOI.

### Statistical analysis

Repeated-measures two-way analysis of variance (ANOVA; approaches × regions) was used to compare means of spatial registration parameters, regional values of SUV and BP_ND_ among approaches, when applicable. When the approach effect was observed, a secondary paired t-test was performed. Linear regression was used to evaluate correlations between two variables, when applicable. Bonferroni correction was applied for all t-tests, for multiple testing across 7 regions and 3 axis directions. All statistical analyses were performed using R Statistical Software (v4.1.2; R Core Team 2021).

## Results

### Effects of initial guesses on coregistration

We observed that initial estimates, as measured by registration errors, varied widely across rater-animal combinations (Fig. 2a). Note that initial guesses of linear and rotational parameters from the PET portion, and scaling parameters from the CT portion of PTCT were used as initial guesses for PT9 and PT12. Despite the variations of initial guesses, PTCT successfully converged for CT and PET for all cases showing registration errors below respective criteria including the two subtle outliers. On the other hand, PT9 and PT12 failed to converge successfully in 4 (10%) or 5 (12.5%) cases respectively (Fig. 2b). These findings suggested that PTCT demonstrated improved reproducibility and accuracy over ill-defined initial guesses than PT9 and PT12 approaches. Since the results successfully converged for PTCT across all users and most users for PT9 and PT12, we present the results obtained with the initial guesses from one rater (AN) whose initial guesses resulted in successful conversions in all animals, to simplify the presentation of results.

#### Comparison of spatial registration parameters:

**Figure 3:**
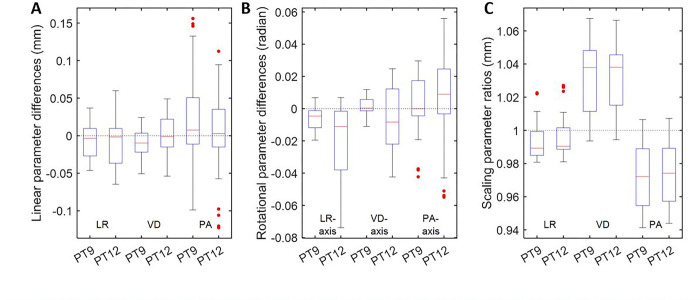
Boxplots of differences of linear (**a**) and rotational (**b**) parameters, and ratios of scaling parameters (**c**) of PT9 and PT12 approaches against proposed PTCT approach. Abbreviations: PTCT = proposed approach; PT9 and PT12 = published approach by 9- or 12-parameter coregistration.; LR = left-to-right; VD = ventral-to-dorsal, and PA = posterior-to-anterior directions or axes.

PT9 and PT12 yielded comparable estimates of linear parameters to PTCT except in the ventral-dorsal direction (PT9 alone; p < 0.0025; t = −3.3; df = 19) albeit negligible differences (range: −0.05–0.03 mm) (Fig. 3). PT9 and PT12 showed lower rotational parameter estimates around the left-right direction (p < 1.2 × 10^− 5^; t < −5.0; middle panel) and higher (p < 3.3 × 10^− 11^; t > 9.1) and lower (p < 1.6 × 10^− 10^; t < − 8.7) scaling factor estimates than PTCT in the dorsal-ventral and posterior-anterior directions, respectively.

### Comparison of PET measures

**Figure 4:**
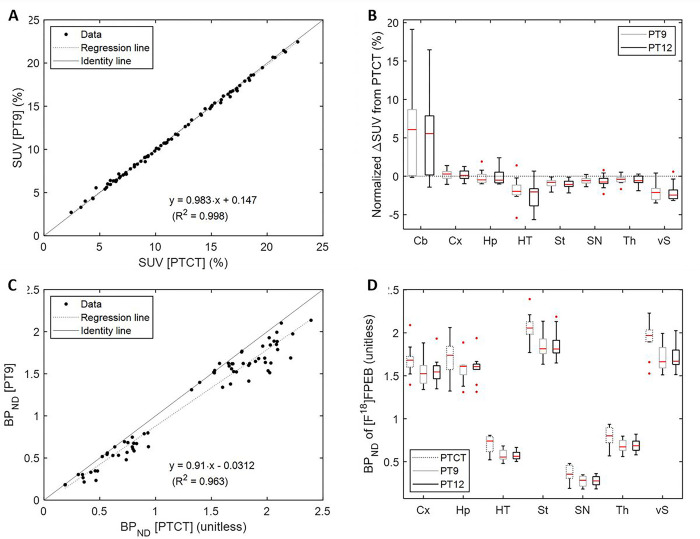
Scatter plots of regional SUV (**a**) and BP_ND_ (**c**) data, PT9 (=y) versus PTCT. Boxplot of normalized ΔSUV (**b**) (PT9 or PT12 less PTCT over PTCT). Boxplot of regional BP_ND_ data for the three approaches (**d**).

Regional SUV values were tightly correlated between PT9 and PTCT (Fig. 4a) and PT12 and PTCT (PT12 = 1.01∙PTCT – 0.091; R^2^ = 0.999). Despite tight correlations, both PT9 (t = 3.53; Bonferroni-adjusted p < 0.01; paired t-test; Fig. 4b) and PT12 (t = 3.18; adjusted p < 0.02) showed greater SUV values in Cb than PTCT while showing lower SUV values in St and HT (t < −3.43; adjusted p < 0.01 for PT9 and PT12) as shown in 4B. Because of underestimation of SUV in Cb, PT9 (Fig. 4c) and PT12 (not shown) showed roughly 10% lower BP_ND_ values across regions than PTCT. Using a paired t-test, we confirmed that PTCT showed higher BP_ND_ values in all brain regions (Fig. 4d) than both PT9 (Bonferroni-adjusted p < 0.001) and PT12 (adjusted p < 0.001). Note that indistinguishable values were observed between PT9 and PT12 for SUV (PT12 = 1∙PT9–0.0002; R^2^ = 0.999) and BP_ND_ (PT12 = 1∙PT9 + 0.002; R^2^ = 0.998) (Table 2).

### Secondary Analyses

**Figure 5:**
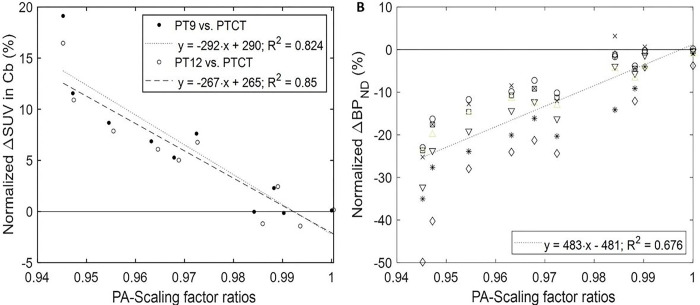
Scatter plots correlating normalized ΔSUV (PT9 or PT12 less PTCT over PTCT) in the cerebellum, Cb (**a**) and normalized ΔBP_ND_ in the target regions (B for PT9; y = 451×x − 450; R^2^ = 0.642 for PT12) to the ratios of posterior-anterior (PA) scaling parameters to PTCT. Symbols for the regions in (**b**) were o: cortex; x: hippocampus; *: hypothalamus; □: striatum; ◊ substantia nigra; ▽: thalamus; and Δ ventral striatum.

We examined whether differences of SUV and BP_ND_ estimates, given by normalized ΔSUV and Δ BP_ND_ (PT9 or PT12 less PTCT over PTCT) were driven by the differences of scaling factor estimates, given by scaling factor ratios (PT9 or PT12 over PTCT). Normalized ΔSUV linearly correlated to LR-scaling factor ratios in Hp (positive), to DV-scaling factor ratios in HT, Th, and vS (all negative), and to PA-scaling factors in Cb (negative) (Bonferroni-corrected p < 0.03 for 8 regions and 3 axes directions). Note that normalized ΔSUV reached up to 20% in Cb (Fig. 5a) while remaining up to 5% in other regions. Normalized ΔBP_ND_ positively correlated to PA-scaling factor ratios when all regions were pooled (Fig. 5b) or in individual regions (Bonferroni-corrected p < 0.01 for 7 regions and 3 axes directions).

**Figure 6:**
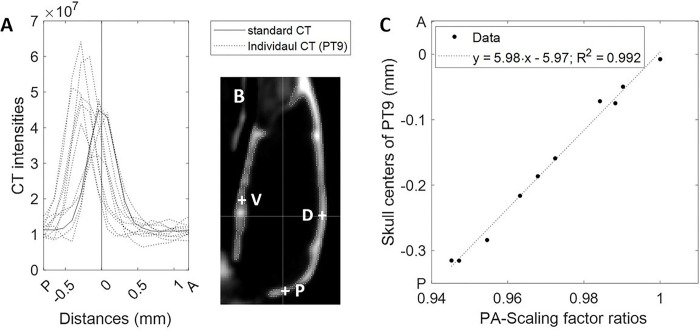
Line plots of CT intensities of individual CT images in the standard space using scaling factor estimates from PT9 (**a**) along the posterior-anterior axis at the posterior point P in (**b**). Other evaluation points, the ventral (V) and dorsal (D) points are also shown in (**b**). Scatter plots correlating the skull centers of individual animals at P shown in A to the scaling factor ratios in the posterior-anterior direction (**c**)

Evaluation of CT intensity profiles at the five ‘perpendicular’ skull points revealed no deviations of individual skull centers by PTCT (**Supplemental Fig. 1**). However, various degrees of deviations were observed for individual CT images (**Supplemental Fig. 2**) that were transferred using scaling factor estimates from PT9 (Fig. 6a, b). As expected, deviations of the skull centers were proportional to scaling factor ratios at all ‘perpendicular’ skull points (Bonferroni-corrected p < 6×10^− 8^ for the 5 skull points) as exemplified in Fig. 6C for the posterior point, except at the ventral point (Fig. 6b, c). When the observation was extended to all voxels within the skull VOIs, CT images derived from the scaling factor estimates of PT9 showed lower CT intensities than CT images from PTCT in all sextant divisions of the skull VOI (**Supplemental Fig. 3a-c**). Altogether, the examinations of spatial agreement of the skull indicated that PT9 could yield varying degrees of errors in the scaling factors, especially in the PA direction.

### Statistical Power

To assess the power achieved in the statistical comparison of PTCT vs PT9/PT12-derived estimates, we estimated an error of 10% for PTCT and a 12–16% error for PT9 and PT12 approaches, based on the additional “error” on the PT9 / PT12 side present in the data. Based on these assumptions the analysis of n = 16 subjects achieved > 90% for PTCT, and 85% power for PT9/PT12, to detect group differences (independent samples t-test) of at least 10% in BP_ND_. Our analysis was slightly underpowered (achieved power 75% for PTCT, 70% for PT9/PT12) to detect smaller changes of at least 5%. In terms of detecting within-group differences (repeated measures t-test), we had slightly greater power: for PTCT, 95% power achieved for differences of 10% or greater but 80% power for smaller differences of 5%. Finally, power was slightly lower for PT9/12, at 90% and 75% for 10% and 5% differences, respectively.

## Discussion

The novelty of the proposed PTCT lies in the use of CT-derived estimates of the scaling factors for customization of the standard PET volumes to match the dimensions of individual animals’ brains. These measures made spatial registration of PET volumes robust. In contrast, conventional PT9 [11, 22, 23] and PT12 [8, 24] approaches estimated brain dimensions solely with PET images.

In support of PTCT, this study presented compelling evidence that PET-based approaches could not estimate the brain dimensions of individual animals accurately for ^[18^F]FPEB scans from the following observations: First, CT intensity profiles of the skulls of individual animals agreed with the standard CT for PTCT but shifted in various degrees among animals with the scaling factor estimates from PT9. Second, skulls of individual animals aligned better with the standard skull for PTCT than for PT9-derived scaling factor estimates.

In this study, the PET-based approaches (PT9 and PT12) showed various degrees of underestimations of scaling factors in the anterior-posterior direction compared to PTCT. This resulted in negatively proportional overestimation of radioactivity in Cb up to 20%, and proportionally more prominent underestimation of BP_ND_ in target regions down to −50% (but − 10% on average). The latter findings could be due to combined effects of the underestimations of scaling factors in the anterior-poster direction paired with smaller under-/over-estimation in other directions.

The results from PTCT showed better agreement with the biological expectations of the receptor system than PT9 or PT12. First, PTCT yielded lower radioactivity in Cb where mGlu5Rs have negligible expression in rodents [34, 42]. Second, PTCT showed higher SUV values in known target regions with higher expression of mGluR5 [42]. Altogether, this study demonstrated that PTCT improved special registration of PET images and accuracies of PET outcome variables over the conventional PET-based approached of PT9 and PT12 for [18F]FPEB scans done with the PET-CT scanners.

It should be emphasized that PTCT does not require special registration of PET and CT images for individual scans. Interestingly published CT-based approaches [13–15] assumed intrinsic alignment of CT and PET volumes (i.e., no head motion between CT and PET acquisitions). Accordingly, no PET-CT alignment approaches were provided in these papers. In contrast, we detected head motion between PET and CT scans in most animals in this study on visual inspection. We also experienced that SPM12’s coregistration code rarely produced acceptably confident CT-PET registration results. Therefore, PTCT, which is unaffected by head motion between CT and PET scans, could be advantageous over existing CT-based approaches.

In this study with [18F]FPEB, we demonstrated that PT9 and PT12 underestimated regional BP_ND_ in varying degrees across animals, and overall changed values by ~ 9–10%. Therefore, PT9 or PT12 could suffer additional variability of observed BP_ND_ values due to erroneous coregistration estimates. As a future approach, we plan to compare PTCT vs PT9 and PT12 across more tracers, to strengthen findings from this study in support of PTCT over PT9 or PT12.

The coregistration code of SPM12 provided the algorithm for CT-to-CT and PET-to-PET coregistration procedures. In theory, it was possible to include parameters for shear (as in PT12) in PTCT owing to the SPM module’s ability to accept rearrangement of parameter orders (i.e., linear, rotational, and shear, skipping scaling). However, we did not find any need for the addition because the PT9 and PT12 approaches yielded practically identical estimates of spatial registration parameters, regional radioactivity values and BP_ND_ values. We also evaluated incorporating spatial normalization in PTCT since spatial normalization of individual PET to standard PET is also reported for rodent PET studies [8, 11, 12, 21, 24, 43, 44]. We could not obtain acceptable convergence on visual inspection for normalization of the final products of PTCT (PET images transferred to the standard PET) to the standard PET of [18F]FPEB despite trying various published approaches and our own fine tuning of estimation parameters of the normalization code of SPM12.

We found that none of the tested approaches converged acceptably well without supplying the initial guesses of parameters, potentially due to the skewed head positions of the animals in the scanner to allow scanning two animals at a time without restricting breathing. In this study, the raters were instructed to obtain ‘rough’ estimates of initial guesses to evaluate dependency of automated refinements on initial guesses. The evaluation results indicated less dependency of PTCT than PT9 and PT12 and emphasized the importance of implementing the quality control process of the automated refinements. As to the approaches for initial guesses, we would like to emphasize that the proposed approach (i.e., to displace images to align to the standard CT or PET outlines) which has not been published to our knowledge made it significantly easier for the raters to grasp spatial orientation than the opposite approach (i.e., to displace the standard CT or PET outlines to align to images).

### Limitations

First, PTCT may be applicable to scans from PET-CT scanners alone unless CT images are obtained separately. Nevertheless, PTCT will continue to be useful due to current and continued dominance of PET-CT scanners over emerging PET-MRI scanners owing to oncology applications [44, 46–48]. Second, PTCT may be applicable to conditions in which the normal skull-brain relationships are maintained. Analyses with anatomical scan (i.e., MRI) may be required for conditions that could accompany alterations to the normal brain anatomy such as cases involving bleeding, localized edema (e.g., head trauma), etc. Third, PTCT solely relies on linear spatial registration. PET-based non-linear coregistration approaches failed to converge reasonably well in this study (data not shown) even using personalized standard [18F]FPEB volumes. In contrast, promising results were published for MRI-based non-linear coregistration approaches [49–51]. Nevertheless, these publications failed to critically compare MRI-based and conventional PET-based approaches.

Additionally, PTCT has been evaluated with scans of [18F]FPEB alone for now, yet from our results it appears reasonable to assume PTCT as the first choice for quantitative analyses of PET data of any radiotracer from PET-CT scanners since the CT-based scaling factors underpinned the core improvement of PTCT over the PET-based PT9 and PT12 approaches. Nevertheless, we plan to comparatively evaluate PTCT with other PET tracers to make the conclusions from this study more concrete.

Therefore, future directions may include evaluation of PTCT against MRI-based approaches from PET-MRI scanners to address whether non-linear spatial registration is indispensable for rodents as well. Cross-validation is needed especially because this study demonstrated that minor differences in spatial registration could result in notable differences in SUV and BP_ND_ values.

### Conclusions

We demonstrated that PTCT, which uses CT-derived scaling factors, could be an improvement over conventional PET-based coregistration approaches, such as PT9 and PT12 which suffered various degrees of errors in scaling factor estimates. In [18F]FPEB scans, these errors most affect the reference region (Cb), leading to underestimation of regional estimates of the PET outcome variable, BP_ND_ compared to PTCT. As it is expected that PET-CT scanners will continue to be widely used and PTCT is a novel co-registration approach that improves estimates of BP_ND_ in [18F]FPEB scans, future planned studies include investigating PTCT with other radiotracers and validation against PET/MRI scanners. These directions will determine the extent of the utilization of PTCT, as it is expected that PET-CT scanners will continue to be widely used.

## Figures and Tables

**Table 1:** Volume and Scaling factors, by VOI

Regions:	volumes (mL)	Left-Right direction (mm)	Dorsal-Ventral direction	Anterior-Posterior direction
left cortical mantle	0.03	4.39	3.53	8.53
right cortical mantle	0.03	4.26	3.66	8.41
left striatum	0.01	2.44	2.19	3.78
right striatum	0.01	2.44	2.32	3.78
left ventral striatum	0.00	0.61	0.37	0.49
right ventral striatum	0.00	0.49	0.37	0.49
left hippocampus	0.01	3.53	3.53	3.05
right hippocampus	0.01	3.17	3.78	2.80
left thalamus	0.01	2.07	2.07	3.17
right thalamus	0.01	2.19	2.07	3.17
left hypothalamus	0.00	1.95	2.19	3.53
right hypothalamus	0.00	1.95	1.95	3.90
left substantia nigra	0.00	0.97	1.10	1.22
right substantia nigra	0.00	0.97	0.97	1.22
cerebellum	0.03	7.68	4.51	3.66

**Table 2:** Statistics for comparisons of PT9 vs PT12 vs PTCT methods, using the total mean deviation from the standard.

Directions	Regions	Approaches	Regression	R^2^	p-value
**Left-right**	**Hippocampus**	**PT9 vs. PTCT**	**y = 71⋅x - 71**	**0.983**	**2.49⋅10^−8^**
		**PT12 vs. PTCT**	**y = 78⋅x - 78**	**0.949**	**2.10⋅10^−6^**
**Dors al-Ventral**	**Hypothalamus**	**PT12 vs. PTCT**	**y = −70⋅x + 71**	**0.545**	**4.28⋅10^−4^**
	**Ventral striatum**	**PT9 vs. PTCT**	**y = −54⋅x + 54**	**0.845**	**1.67⋅10^−4^**
		**PT12 vs. PTCT**	**y = 56⋅x + 55**	**0.858**	**6.29⋅10^−4^**
**Anterior-Posterior**	**Cerebellum**	**PT9 vs. PTCT**	**y = −292⋅x + 290**	**0.824**	**2.86⋅10^−4^**
		**PT12 vs. PTCT**	**y = −267⋅x + 265**	**0.850**	**2.51⋅10^−4^**

## Data Availability

The datasets used and/or analyzed during the current study are available from the corresponding author on reasonable request.

## Abbreviations

### Regions:

Cb: cerebellum
Cx: cortical mantle
Hp: hippocampus
HT: hypothalamus
St: Striatum
SN: substantia nigra
TH: thalamus
Vs: ventral striatum: Vs

### Methods:

PTCT: Proposed CT-based coregistration Approach
PT9: PET-based rigid body coregistration with 9 parameters
PT12: PET-based affine coregistration with 12 parameters

### Variables:

BP_ND_: binding potential, non-displaceable
SUV: standardized uptake value
ΔSUV: normalized difference in SUV
Δ BP_ND_: normalized difference in binding potential
